# Factors Associated with the Rapid and Durable Decline in Malaria Incidence in El Salvador, 1980–2017

**DOI:** 10.4269/ajtmh.17-0629

**Published:** 2018-05-14

**Authors:** Robert A. Burton, José Eduardo Romero Chévez, Mauricio Sauerbrey, Caterina Guinovart, Angela Hartley, Geoffrey Kirkwood, Matthew Boslego, Mirna Elizabeth Gavidia, Jaime Enrique Alemán Escobar, Rachel Turkel, Richard W. Steketee, Laurence Slutsker, Kammerle Schneider, Carlos C. (Kent) Campbell

**Affiliations:** 1 Center for Genomic Interpretation, Sandy, Utah;; 2 Ministerio de Salud, San Salvador, El Salvador;; 3 The Carter Center, Atlanta, Georgia;; 4 PATH Malaria Control and Elimination Partnership in Africa (MACEPA)/ISGlobal Collaboration, Barcelona Institute for Global Health (ISGlobal), Barcelona, Spain;; 5 PATH, Seattle, Washington

## Abstract

A decade after the Global Malaria Eradication Program, El Salvador had the highest burden of malaria in Mesoamerica, with approximately 20% due to *Plasmodium falciparum*. A resurgence of malaria in the 1970s led El Salvador to alter its national malaria control strategy. By 1995, El Salvador recorded its last autochthonous *P. falciparum* case with fewer than 20 *Plasmodium vivax* cases annually since 2011. By contrast, its immediate neighbors continue to have the highest incidences of malaria in the region. We reviewed and evaluated the policies and interventions implemented by the Salvadoran National Malaria Program that likely contributed to this progress toward malaria elimination. Decentralization of the malaria program, early regional stratification by risk, and data-driven stratum-specific actions resulted in the timely and targeted allocation of resources for vector control, surveillance, case detection, and treatment. Weekly reporting by health workers and volunteer collaborators—distributed throughout the country by strata and informed via the national surveillance system—enabled local malaria teams to provide rapid, adaptive, and focalized program actions. Sustained investments in surveillance and response have led to a dramatic reduction in local transmission, with most current malaria cases in El Salvador due to importation from neighboring countries. Additional support for systematic elimination efforts in neighboring countries would benefit the region and may be needed for El Salvador to achieve and maintain malaria elimination. El Salvador’s experience provides a relevant case study that can guide the application of similar strategies in other countries committed to malaria elimination.

## INTRODUCTION

The malaria program in El Salvador is currently in the elimination stage, with only four cases of *Plasmodium vivax* reported in 2017 among its population of 6.1 million (three imported, one relapse from 2016) (E. Romero, personal communication). The last case of locally transmitted *Plasmodium falciparum* in El Salvador was recorded in 1995 and the last death from *Plasmodium* infection occurred in 1984.^1^ In 1980, the country contributed 37% of all reported cases in the region, whereas today it contributes less than 0.1%.^2^

**Figure 1.**
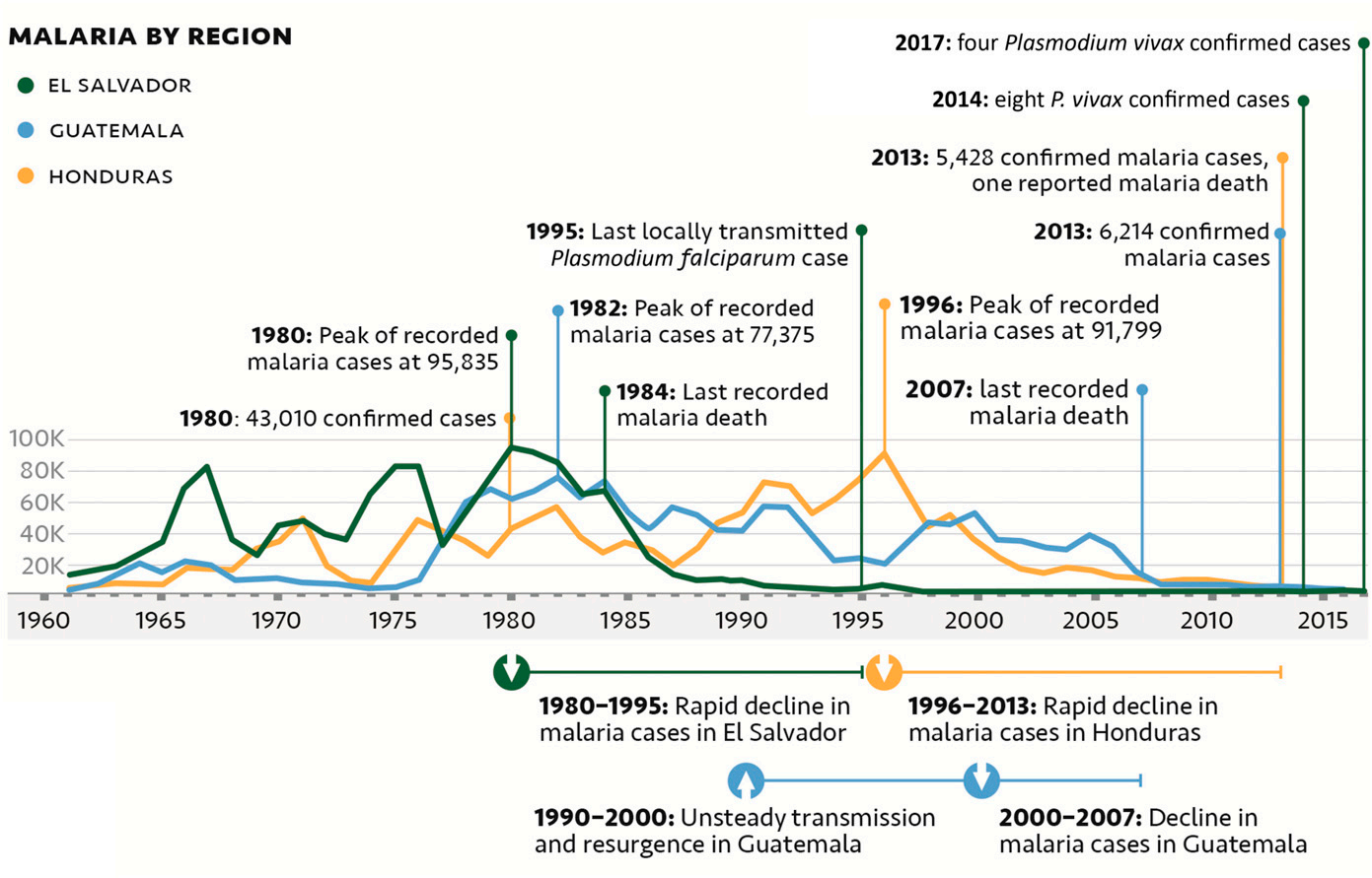
Annual malaria cases by country and national benchmarks in the decline of malaria in the region from 1961 to 2017.^2^

**Figure 2.**
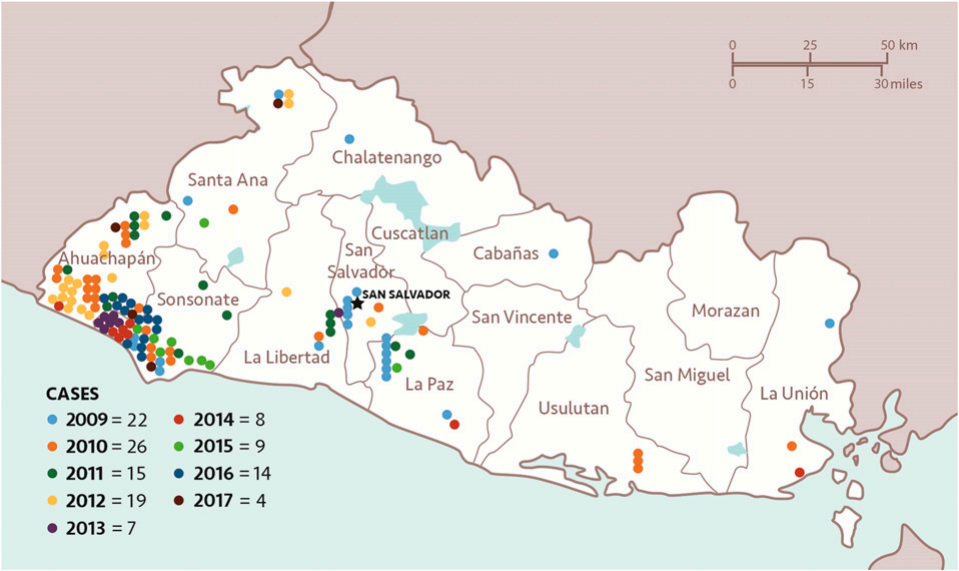
All reported (imported and autochthonous) malaria cases in El Salvador, 2009–2017 (National Vector Control Program, personal communication).

El Salvador is socioeconomically and epidemiologically similar (in terms of malaria) to its Mesoamerican border neighbors: Guatemala, Honduras, and Nicaragua (Table 1). Key differences include the extent of urbanization and the overall improved quality of the health system in El Salvador.^3^ For most of its recent history, patterns of malaria transmission in the country were analogous to those in the region: periods of decline in incidence followed by periods of resurgence (Figure 1).^2^ Beginning in the early 1980s, the number of cases detected each year began to decline, with a 90% reduction in cases occurring between 1980 (95,835 reported cases) and 1990 (9,269 reported cases).^2^ El Salvador continued to reduce locally acquired cases each year as remaining transmission foci were cleared, with less than 50 cases annually since 2006; by contrast, its immediate neighbors continue to have the highest incidences of malaria in the region (Table 2, Figure 2).^2^

**Table 1:** Similarities and differences between El Salvador and its Mesoamerican neighbors.^3–7^

Similarities	Description	El Salvador	Guatemala	Honduras	Nicaragua	Mean ± 1 SD
Topographical and ecological	Low-land coasts with tropical climate	✓	✓	✓	✓	–
	Interior highlands with temperate climate	✓	✓	✓	✓	–
	Primary vectors: *Anopheles albimanus*, *Anopheles darlingi*, *Anopheles pseudopunctipennis*	✓	✓	✓	✓	–
	Transmission highest during the rainy season (May to October)	✓	✓	✓	✓	–
Socioeconomic	Low per capita gross domestic product (2015) in $US (rank relative to the rest of North America)	$8,300 (19/23)	$7,900 (20/23)	$5,000 (21/23)	$5,000 (22/23)	$6,550 ± $1,797
	% Reduction in export of cotton (1977–1992)	↓95.0%	↓85.7%	↓84.6%	↓98.1%	↓90.9% ± 6.7%
	% Increase in population density (1961–2013)	↑323%	↑291%	↑361%	↑332%	↑327% ± 29%
Geopolitical	Civil war	1980–1992	1960–1996	–	1978–1990	–
Differences*						
Topographical and ecological	Total land area (km^2^)	20,721	107,159	111,890	120,340	90,028 ± 46,525
Socioeconomic	Total population density (2014) (people/km^2^)	295	149	71	50	141 ± 111
Health indicators	Under-five mortality per 1,000 live births (2013)	16	31	22	24	23 ± 6
	Maternal mortality per 100,000 live births (2015)	54	88	129	150	105 ± 43

SD = standard deviation.

* El Salvador data fall more than one SD away from the mean.

**Table 2:** Number of annual reported malaria cases per country in Mesoamerica

Country	Year											
	1960	1965	1970	1975	1980	1985	1990	1995	2000	2005	2010	2015
El Salvador	**10,066**	**34,070**	45,436	**83,100**	**95,835**	44,473	9,269	3,364	753	67	24	9
Guatemala	3,387	14,472	11,044	4,979	62,657	54,958	41,711	24,178	**53,311**	**39,571**	7,198	**5,540**
Honduras	5,517	6,952	34,537	30,289	43,010	33,828	**53,099**	**74,346**	35,125	16,007	**9,745**	3,575
Mexico	3,569	10,113	**61,158**	27,925	25,734	**133,698**	44,513	7,423	7,390	2,967	1,233	551
Nicaragua	7,528	10,275	27,260	24,692	25,465	15,130	35,785	69,444	23,878	6,642	692	2,307
Costa Rica	2,000	2,563	350	290	376	734	1,151	4,515	1,879	3,541	114	8
Panama	4,464	1,929	4,584	666	304	126	381	730	1,036	3,667	418	562
Belize	196	206	33	90	1,529	2,800	3,033	9,413	1,486	1,549	150	13
Total cases	36,727	80,580	184,402	172,031	254,910	285,747	188,942	193,413	124,858	74,011	19,574	12,565
Contribution (%): El Salvador	27.41	42.28	24.64	48.31	37.60	15.56	4.91	1.74	0.60	0.09	0.12	0.07

The bolded numbers in each column identify the country with the highest number of cases in a given year.^2^

Lessons learned from El Salvador’s success could guide malaria elimination strategies in similar countries. We conducted a review and evaluation of the policies and interventions implemented by the Salvadoran National Malaria Program (NMP) to document the progress and assess factors that likely contributed to this success.

## MATERIALS AND METHODS

A systematic approach to the acquisition and analysis of malaria data and associated public health data was adopted for this case study.^8–10^ Data from peer-reviewed and gray literature—as well as records and documentation from district-level and national malaria efforts—were obtained and compiled. Substantial gaps in publicly available literature and databases were identified. Additional information detailing specific program strategies, intensity of activities, treatment guidelines, surveillance systems, stratification methodologies, and programmatic timelines were compiled through a series of interviews with local and regional malaria and vector control experts.

More than 27 experts from 16 international organizations and national and regional institutions from El Salvador, Guatemala, and Honduras were interviewed as part of this research. These sources were selected based on their expertise and knowledge of current and historical malaria epidemiology and government programs in the region. Individual contributors and their affiliations are listed in the Acknowledgments section.

Review of the literature and interview data resulted in a detailed timeline and comparative analysis of possible factors contributing to the decline in El Salvador’s malaria incidence. These data also helped generate hypotheses as to which factors were key contributors to the initial decline and which factors had contributed to the maintenance of low malaria incidence.

## RESULTS

The period of rapid decline in El Salvador’s malaria incidence began with a deliberate program transition following an evaluation of the national malaria control strategy in partnership with the Pan American Health Organization (PAHO) and the United States Centers for Disease Control’s (CDC) local research station in 1978.^11,12^ The objective of this evaluation was to course correct the program after a dramatic increase in cases in the late 1970s, a decade after the global malaria eradication campaign ended.^11,12^ The evaluation examined data from the previous 7 years of malaria control activities, developed a mostly geographic stratification system by malaria risk, and used it to inform program strategy and resource allocation (E. Romero, personal communication).

The malaria program was decentralized and the new approach was focused on a well-functioning national surveillance and reporting system, as well as on evidence-based targeted and timely responses to case detection conducted weekly. Successful decentralization in El Salvador was facilitated through the appointment of local leaders who were given authority to act in real time and who were held responsible for progress in their respective areas. In 1980, a PAHO evaluator summarized the new malaria program in El Salvador as a streamlined process in which “the data are obtained in the shortest possible time and a dynamic and timely epidemiology can be conducted in line with the new orientation of the program.”^13^ These concepts were novel at the time of introduction and contributed to reducing malaria incidence during the 1980s and 1990s.

As cases dropped to very low levels, stratification continued to drive prioritization of activities; vector control interventions were increasingly targeted, border surveillance of immigrants was launched to address imported cases, prompt diagnosis was maintained, and the surveillance system continued to track malaria cases.

### Malaria risk stratification.

**Figure 3.**
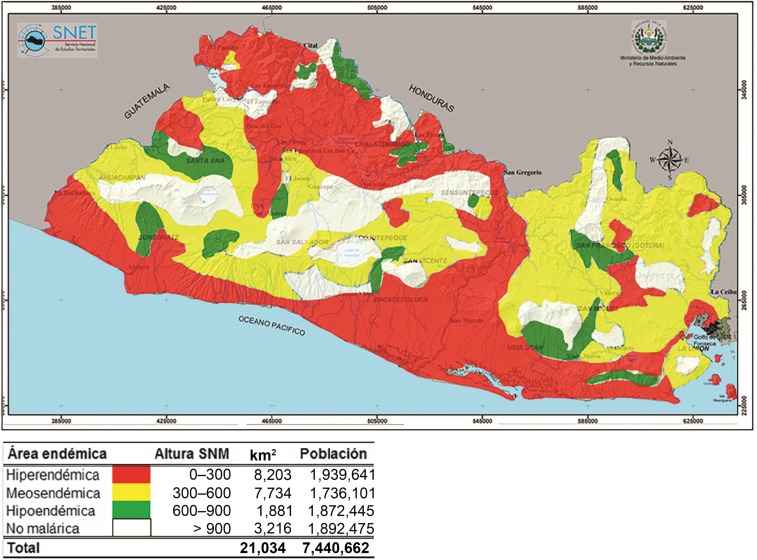
Topographical map of El Salvador detailing the results of the malaria epidemiological stratification (National Vector Control Program, personal communication). Regions of hyperendemicity (hiperendémica), moderate endemicity (mesoendémica), and hypoendemicity (hipoendémica) are shown in red, yellow, and green, respectively. Areas with no malaria endemicity (no malárica) are shown in white. The altitude above sea level (altura sobre el nivel del mar [SNM]), total land area in km^2^, and population (población) of each region are also indicated in the key below the figure.

The El Salvador NMP worked to determine the geographic distribution and frequency of malaria cases, with the objective of stratifying the country by risk based on altitude and the number of blood smear–confirmed cases per 1,000 residents (M. Sauerbrey, unpublished data).^14^ With the support of United States Agency for International Development and CDC, epidemiologic stratification was completed by 1979 (Table 3, Figure 3) and these data were then used to transition and target program activities.^15^ Placement and number of volunteer collaborators (VCs), number of laboratory and supervisory personnel, and vector control activities were all determined by strata, enabling the highest burden areas to have proportionately higher resources. By 1980, stratification was an integral part of the malaria strategy and has remained so (J. Alemán, personal communication).

**Table 3:** Original CDC-CAR malaria stratification in El Salvador, 1979^12^

Strata	Area square kilometer (%)	Total cases (%)	Number of VC posts (%)	Population (%)
Non-malarious (0–19 cases/year) (> 901 m)	1,888 (9%)	1	382 (13%)	538,979 (10%)
Hypoendemic (20–39 cases/year) (601–960 m)	11,118 (53%)	3	499 (17%)	3,395,567 (63%)
Mesoendemic (40–59 cases/year) (301–600 m)	3,216 (15%)	6	656 (23%)	485,081 (9%)
Hyperendemic (> 60 cases/year) (0–300 m)	4,819 (23%)	90	1,377 (47%)	970,162 (18%)

CDC = Centers for Disease Control; VC = volunteer collaborator.

### Voluntary collaborator network.

The VC network, a vertical community malaria platform, was established in El Salvador in the 1950s to test and treat all febrile individuals with antimalarials. It eventually grew from 79 posts in 1955 to 590 posts in 1959 (M. Sauerbrey, unpublished data). Further expansion, strengthening, and more effective distribution of the VC network resulted from the 1978 decentralization and stratification. Analysis of the annual parasite index (API) and slide positivity rate data obtained before 1978 indicated that approximately 80% of the VCs were located in areas with little or no malaria burden.^13^ Under stratification, the number of VCs in each epidemiological stratum was determined according to malaria risk, with the highest number of VCs deployed to hyperendemic regions (Table 3). The goal was to have one VC for every 600 individuals in the two highest burden strata.

El Salvador’s VC network was established with strict expectations: accuracy, timeliness, and respect for authority.^16^ Volunteer collaborators were traditionally selected by respected members of their own communities, and despite the lack of monetary compensation, they earned the respect of their peers and took pride in providing high-quality service (M. Sauerbrey, personal communication). In many instances, retiring VCs would pass on their duties to other capable family members (M. Gavidia, personal communication).^17^

Volunteer collaborators collected blood smears, provided presumptive antimalarial treatment of all fever cases, and kept records of all fever cases and blood smear laboratory results in the communities they served, although exact historical responsibilities and drug regimens remain unclear. Each VC was assigned a unique identification code so that reported cases could be tracked by geographic area according to VC placement, and all case data were reported to the NMP (E. Romero, personal communication; M. Sauerbrey, unpublished data). As a component of decentralization, VCs worked collaboratively with epidemiology assistants, entomology assistants, and zonal epidemiological surveillance leads.

Epidemiology assistants would visit each of the VCs in epidemiological circuits to collect the blood smears and the surveillance records and to restock VCs with drugs and blood sampling materials, including microscope slides. Entomology assistants were responsible for entomological data collection and larval control activities (M. Sauerbrey, personal communication). The objective of the epidemiological circuits was to provide increased oversight to VCs in areas of highest endemicity. Frequency of epidemiology assistant visits to VCs varied by region and was determined by strata: visits were weekly, biweekly, monthly, or every 3 months, depending on the disease burden.^14^ Epidemiology assistants were also responsible for selecting and training new VCs. Official training varied from 1 to 3 days, and each VC received a box with the register book and all necessary supplies as most of them were working out of their homes (E. Romero, personal communication). In addition to formal training, nearly all VCs benefitted from hands-on training from the previous volunteer, who in most cases was a family member (E. Romero, personal communication; M. Sauerbrey, personal communication).

The VC network continued to operate and grow through the period of the civil war, and remains operative to this day as a core institution of the malaria control efforts in El Salvador. In 2010, 3,246 VCs were reported to be operational.^18^ As an example of the continued importance of the VC network, although only 28% of thick-smear blood slides were taken by VCs in 2011, they detected 47% of malaria cases.^19^ In 2011, presumptive treatment was abandoned, and VCs currently serve a solely diagnostic and surveillance function (E. Romero, personal communication).

As malaria incidence continues to decline, malaria VCs have been integrated into the overall infectious disease control programs in El Salvador and, thus, maintain their malaria diagnostic and surveillance role while expanding their skillset to include other infectious diseases. In 2018, 3,078 integrated VCs were operational across the country.

### Diagnostic laboratory network decentralization.

As a component of the 1978 review, the NMP also recognized the need to decentralize the decision-making and diagnostic laboratory system, increasing the number of local laboratories according to the stratification to shorten slide turnaround time and empowering local decision-making.

This decentralization occurred between 1978 and 1983.^14^ The network of regional laboratories expanded largely along the coast in the highest transmission strata. Whereas some areas continued to struggle with timely turnaround—sometimes 30 days in hypoendemic areas—there was on average a 5-day turnaround in the hyperendemic stratum throughout the 1980s.^12^ Slides were prepared and stored by the VCs and collected by the epidemiology assistants, who transported slides to the laboratories via motorcycle (M. Sauerbrey, personal communication). Increased diagnostic capacity with improved turnaround time enhanced accurate case management and provided a basis for a strengthened information system to make decisions.

### Surveillance and responses.

Beginning in the 1980s, El Salvador reported several malaria indicators on a weekly basis. Epidemiological data were entered manually into the electronic surveillance system to provide the reports via floppy disk or printouts; these were then accessed and evaluated weekly by regional and central managers to guide control efforts.^20^ Weekly reports included number of *P. falciparum* cases, *P. vivax* cases, and blood slides taken by town for the current week, as well as the number for the prior 4 weeks and the yearly total (M. Sauerbrey, personal communication).

Zonal epidemiological surveillance leads were responsible for epidemiological surveillance, administrative tasks, supervision, and, most importantly, planning of control interventions at the local level.^12^ Changes in API compared with the three previous years in the health catchment areas—down to the hamlet level (*caserío* in Spanish)—were reviewed to develop the annual operational planning.^12^ In addition, the epidemiological surveillance leads reviewed data from their area weekly and were empowered to independently respond to observed changes in a timely manner with an array of different interventions, including additional indoor residual spraying, focal or mass administration of antimalarial drugs, and/or application of larvicides (Table 4).^13^

**Table 4:** Interventions applied in combination according to season and local entomological and epidemiological data in the 1980s^13^

Targeted at the vector
Entomological surveillance and mapping of breeding sites
Application of larvicides (chemical products) to breeding sites
Intra- and peri-domiciliary ultralow-volume aerial spraying
Indoor residual spraying in areas with no insecticide resistance
Targeted at the individual
Utilization of radical cure for all cases
Focal or mass drug administration to specific populations (coffee and cotton agricultural workers) or caseríos
Targeted at the environment and the community
Environmental management to reduce breeding sites (build canals, weed water streams, etc.)
Behavior change communication and community engagement

**Figure 4.**
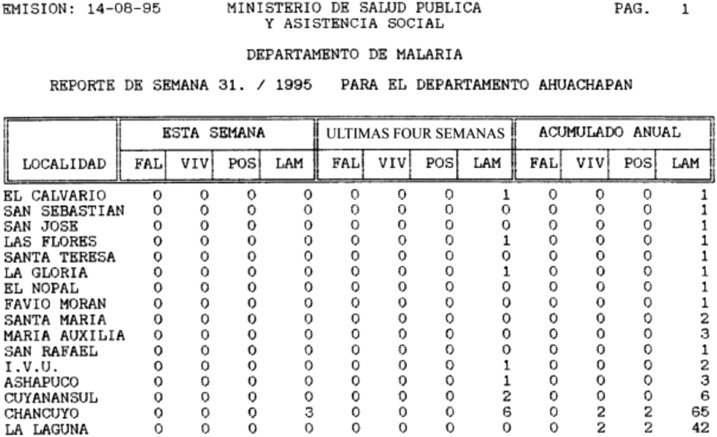
Electronic scan of a portion of a weekly malaria report computer printout from 1995 (M. Sauerbrey, personal communication). The location (localidad), current week (esta semana), last 4 weeks (ultimas four semanas), and cumulative yearly (acumulado annual) totals are shown for *Plasmodium falciparum* (FAL), *Plasmodium vivax* (VIV), the number of positives (POS) determined, and the number of microscope slides taken (LAM).

In 1990, the computerized malaria information system was implemented to address gaps and delays within the manual system and to better track cases, monitor intervention coverage, and supply data on a weekly basis (Figure 4). A USAID evaluation report described the computerization of the information system as necessary to increase the system flexibility and capitalize on recent program successes.^21^ Later, malaria register data began to be reported through the national epidemiological surveillance system, which feeds into the national health information system (J. Alemán, personal communication).

Since 2010, case investigation is conducted when a new malaria case is confirmed to prevent further transmission. The vector control coordinator organizes a response with VCs and local health teams within 24–48 hours of the case confirmation, and focal control actions are implemented (investigating the case’s household, the neighboring households, and other places the case may have visited).^14^ When two or more malaria cases are confirmed, the health facility and vector coordinators follow “outbreak response” guidelines.^19^ Further investigation is conducted to classify cases as either locally acquired or imported based on travel history.^15^ Table 5 shows the number of confirmed autochthonous and imported malaria cases by year between 2010 and 2017 (E. Romero, personal communication).^2^ Of the 14 confirmed *P. vivax* malaria cases in 2016, one was imported and the other 13 could not be investigated because of social risk in the area; of the four *P. vivax* malaria cases in 2017, three were imported and one was a relapse in a case from 2016 (Table 5) (E. Romero, personal communication).^2^

**Table 5:** Classification of malaria cases in El Salvador, 2011–2017 (National Vector Control Program, personal communication)

Vector	2011	2012	2013	2014	2015	2016	2017
*Plasmodium vivax*	15	19	7	8	9	14	4
Autochthonous	9	13	6	6	3	13*	1†
Imported	6	6	1	2	6	1	3
*Plasmodium falciparum*	13	3	0	0	0	0	0
Total recorded cases	25	21	7	8	9	14	4

* Not investigated.

† Relapse.

### Case management and change in treatment regimen.

In the early 1980s, El Salvador’s passive case detection (PCD) system consisted of VCs, health centers, and hospitals, although VCs were the primary source of PCD. Initial treatment of fever cases was presumptive and began the day the malaria blood smear slide was taken; if negative smear results were received before the treatment course was over, treatment was terminated (J. Alemán, personal communication). In addition, active case detection occurred during parasitology surveys, where National Malaria Service workers visited residents in homes, collected blood smears from each individual in the home, and provided presumptive treatment to anyone with recent fever.^21^ By the late 1980s, these malaria surveys were being conducted twice each year in selected villages in high-, moderate-, and low-transmission areas in an effort to measure changes in malaria prevalence that were not normally detectable through the passive surveillance system.^12^ In 1989, VCs were responsible for 70% of all blood samples taken and 94% of all cases diagnosed; 30% of all blood samples taken that year resulted from active case detection.^12^

A 1992 review of the malaria control program activities found that only 4–6% of people who visited VCs with malaria-related symptoms were confirmed to have malaria, suggesting that approximately 95% of patients who did not have malaria received presumptive treatment.^2,12^ This practice continues today, and according to the World Malaria Report, 124,743 courses of treatment were prescribed despite only 21 positive cases recorded in 2012.^22^

Regarding treatment, the NMP transitioned from a 3-day chloroquine (CQ) plus 14-day primaquine (PQ) treatment regimen to a 5-day treatment regimen of combined CQ + PQ in the early 1980s, following a PQ efficacy study conducted in El Salvador by the CDC-Central American Research station.^12,23^ The shortened treatment regimen was intended to address compliance issues of patients not completing the longer treatment course. The program aimed to have all five doses supervised, although it is unclear as to what degree this was completed.

In the early 2010s, following global recommendations and quality control issues found in locally manufactured CQ-PQ, El Salvador changed the treatment regimen to CQ for 3 days followed by PQ for 14 days, and the case management guidelines were changed to treating confirmed cases only (E. Romero, personal communication; J. Alemán, personal communication).^18^ When a case is suspected, a blood smear is taken and analyzed within 24 hours (in the highest transmission strata).^14^ When a positive malaria blood smear is found, the patient must seek treatment from the local health team or facility (VCs do not provide treatment anymore) and then undergo follow-up testing (a blood smear taken monthly for 3 months) after treatment to confirm parasite clearance and conduct surveillance for parasite drug resistance (E. Romero, personal communication).^19^

### Environmental management and vector control.

**Figure 5.**
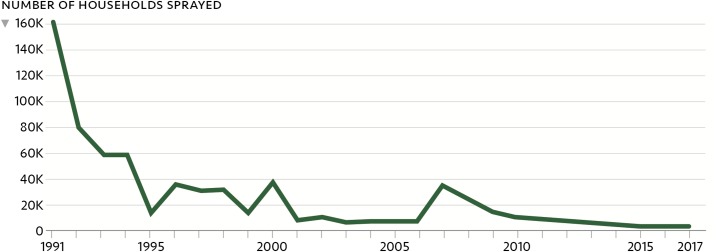
Reduction in the number of households treated with indoor residual spraying as a result of stratification, declining malaria incidence, and environmental projects to improve water management.^2^

The improvement and maintenance of water management projects to eliminate breeding sites were also cited as a key component of El Salvador’s vector control strategy, which aimed to reduce dependence on vector control through indoor residual spraying (Figure 5) (M. Sauerbrey, personal communication).^12^ In the early 1980s, two large environmental management projects began in the Department of La Libertad to limit standing water of two estuaries—areas where the mouths of rivers entering the Pacific Ocean would close during the dry season, producing large mosquito breeding sites often close to large towns (M. Sauerbrey, unpublished data). In total, there were 10 primary source reduction sites, which were the products of collaboration between the National Program, USAID, and PAHO (M. Sauerbrey, personal communication).^12^

### National funding environment.

**Figure 6.**
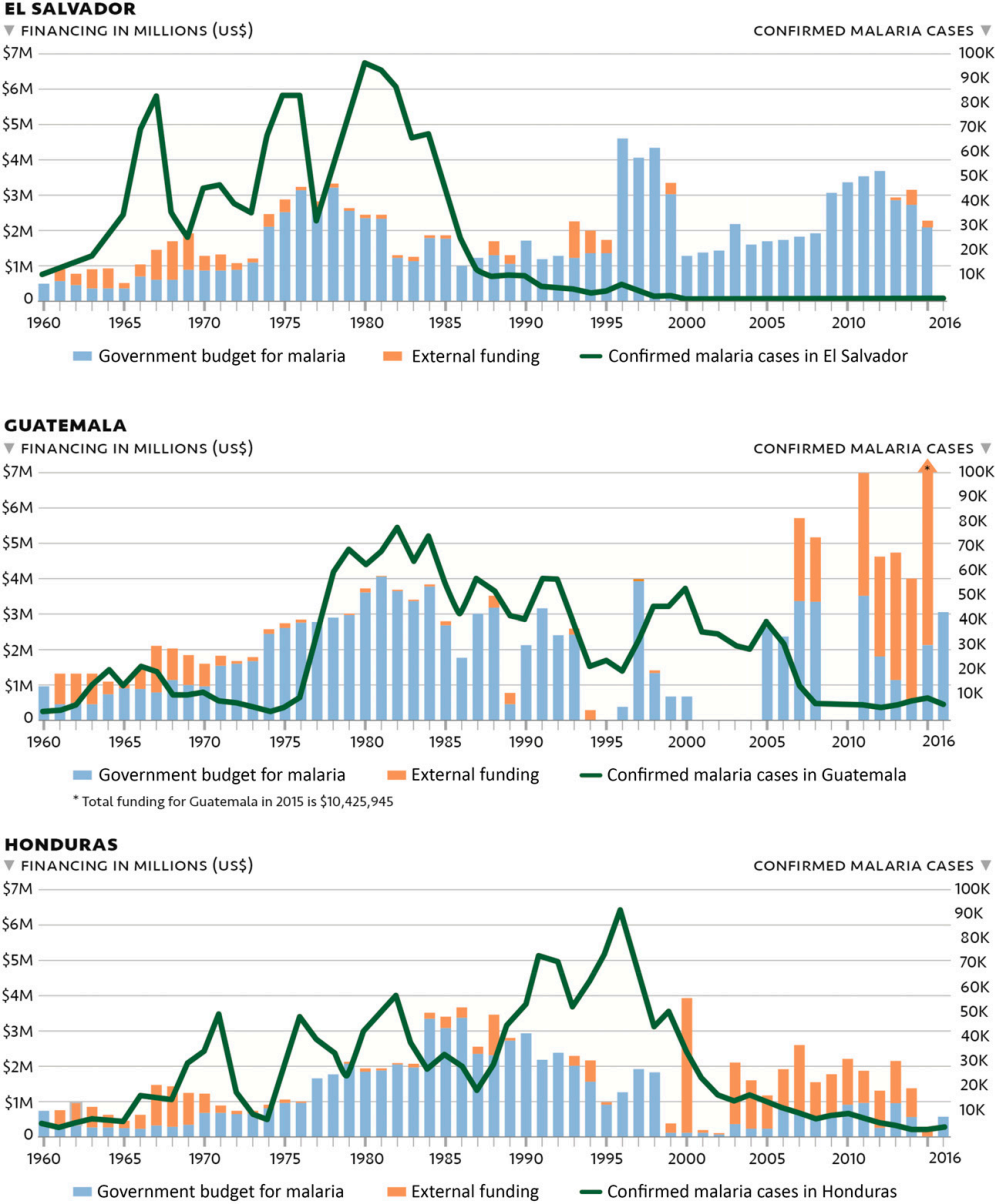
Financing and malaria cases in El Salvador, Guatemala, and Honduras, 1960–2016.^2^

USAID and domestic funding supported activities during the initial phase until the early 1990s, with USAID funding and the CDC research station’s technical support proving critical to the development and implementation of the new strategic plan (M. Sauerbrey, personal communication). However, USAID ceased funding in 1994, leading the national government to increase its financial support to maintain ongoing activities (M. Sauerbrey, personal communication). Starting in 1999, the Ministry of Health distributed the administrative and financial authority of its health system, and the NMP was integrated into the larger National Vector Control Program (E. Romero, personal communication; C. Avila, personal communication).^24^ Despite these structural changes, El Salvador’s malaria activities benefited from stable domestic financing even as external donors withdrew their support (Figure 6).^2^

Furthermore, from 2000 to 2010, as El Salvador achieved very low and continually declining case numbers, domestic financing increased slowly; it was not until 2017 that the government again started receiving external funding for malaria through the Elimination of Malaria in Mesoamerica and Hispaniola Global Fund regional funding mechanism.^25,26^ Currently, domestic funding for national vector control priorities is at the discretion of the Vector Control Program director (E. Romero, personal communication). To date, continued maintenance of the malaria surveillance system and response capacity has been a priority. By contrast, funding for malaria in Guatemala and Honduras has been less predictable throughout the past three decades.

## DISCUSSION

As a model and historic pace-setter, El Salvador offers many lessons to countries in the Mesoamerican subregion and beyond. The NMP in El Salvador undertook a major transition of its program activities in the early 1980s, the key features of which were early stratification, a high coverage of a malaria-specific VC network, targeted allocation of resources, and a strong surveillance system guided by the implementation of a versatile computerized system that allowed precise, data-informed decision-making. Results from this research suggest that El Salvador’s early and continued decline in malaria cases was associated with these interventions and strategies, which were putatively used earlier and more systematically than in Guatemala and Honduras. However, it is probable that socioeconomic factors also played a role. The dramatic decline in malaria cases in the 1980s and 1990s coincided with the Salvadoran civil war (1980–1992), land reform, and the collapse of the national cotton industry. Despite potential interruption of program infrastructure, these factors may have reduced malaria transmission and malaria receptivity by consolidating the population into urban centers, curbing general population movement, and reducing the size of the seasonal cotton workforce at elevated risk of malaria infection (E. Romero, personal communication).

However, in Guatemala and Honduras, where there was also a civil war, a cotton industry that collapsed, and an increase in population density (Table 1), malaria has only recently declined (Figure 1). Taken together, these data suggest that the rapid decline in El Salvador resulted both from NMP actions and from changes in malaria transmission dynamics created by changing socioeconomic conditions. After 1980, El Salvador contributed a continuously decreasing percentage of the regional malaria cases even though overall reported case numbers remained high. Persistent high levels of reported cases among El Salvador’s closest neighbors, Guatemala and Honduras, relative to the region as a whole support the assertion that programmatic actions in El Salvador had a profound effect on malaria incidence and that the decrease cannot be solely attributed to economic development, climate, or ecology. In addition to its smaller size and more dense population, El Salvador’s malaria program benefitted from strong national leadership that ensured sustained financing for malaria control and technical assistance through the in-country presence of the CDC regional research station, followed by a large USAID assistance project and high coverage of the motivated VC network in a vertical program. As stated by Randall M. Packard, “the success of malaria control in El Salvador during the 1980s needs to be viewed as the result of an efficiently designed malaria control program operating within a favorable social and economic environment.”^27^

El Salvador’s multi-decade malaria effort is an impressive success story. It demonstrates that targeted, evidence-based action carried out through a highly organized (yet decentralized) and sufficiently funded program is a successful infectious disease elimination model that other countries or regions may choose to implement. Currently, the importation and spread of malaria from neighboring countries, risk of waning financial support, and loss of programmatic expertise as malaria experts retire and institutional memory and capacity are lost pose the greatest potential challenges for El Salvador to reach the national goal of elimination by 2020. Additional support for systematic elimination efforts in neighboring countries would benefit the region and may be needed for El Salvador to achieve and maintain malaria elimination. Lessons learned from El Salvador’s success can help guide malaria elimination strategies in the region and beyond.
